# Supplementary material for: Open-source interactive design platform for 3D-printed microfluidic devices
Source: Commun Eng. 2024 May 18;3:71. doi: 10.1038/s44172-024-00217-0 (PMC11102439; doi:10.1038/s44172-024-00217-0)
Supplement: Supplementary file 2 — Supplementary Information [file 44172_2024_217_MOESM2_ESM.pdf]

Supplementary Information

Open-Source Interactive Design Platform for 3D-Printed Microfluidic Devices

Yushen Zhang, Mengchu Li, Tsun-Ming Tseng & Ulf Schlichtmann

Supplementary Note 1: Printer and Slicing Settings

Printer Specifications:

Printer model: Anycubic Photon D2
Technology: DLP
Wavelength: 405 nm
Exposure area: 95.42 cm^2
Uniformity: 92%
Light transmittance: 92%
Projector resolution: 2560*1440 px
Layer height: 10 microns
XY resolution: 51 microns
Release film: FEP

Resin:

Model: BV-007A
Manufacturer: Miicraft

Parameters applied for all prints shown in the paper:

Layer thickness: 0.05 mm
Normal exposure time: 3.5 seconds
Off time: 1 second
Bottom exposure time: 28 seconds
Bottom layers: 3
Anti-aliasing: 1
Printer control setting: Basic
Z-axis lift height: 5 mm
Z-axis lift speed: 2 mm/s
Z-axis retract speed: 3 mm/s

Supplementary Note 2: Additional Validation Experiments

In addition to the DLP printer, the Anycubic Photo D2, and BV-007A resin mentioned earlier, we conducted additional tests on the Anycubic Photo D2 using two other different resins, as well as on a MSLA printer, the Elegoo Mars 4, with all three resins to validate Flui3d's practicability across different printing technologies and resin types:

Printer Specifications:

Printer model: Elegoo Mars 4
Technology: MSLA
Wavelength: 405 nm
Exposure area: 99.20 cm^2
Uniformity: 92%
Light transmittance: 95%
Projector resolution: 8520*4320 px
Layer height: 2 microns
XY resolution: 18 microns

Resin:

Model: Plant Based + Clear
Manufacturer: Anycubic

Model: Bio-Med Clear
Manufacturer: Liqcreate

The following table shows the settings and results of each resin and printer combination:

Printer[1]	Resin[2]	Printer and Slicing Setting	DFM Setting	Result (Overview/Close Up)
D2	PB	**Layer thickness:** 0.05 mm<br>**Normal exposure time:** 3.2 s<br>**Off time:** 1 s<br>**Bottom exposure time:** 28 s<br>**Bottom layers:** 2 + 1 transition<br>**Z-axis lift height:** 5 mm<br>**Z-axis lift speed**: 50 mm/min<br>**Z-axis retract speed:** 180 mm/min	**Compensation type:** Local<br>**Compensation min.:** 100 µm<br>**Z level min.:** 200 µm<br>**Compensation max.:** 400 µm<br>**Z level max.:** 1900 µm	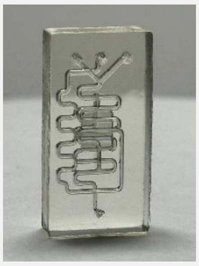<br>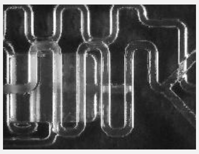
D2	BM	**Layer thickness:** 0.05 mm<br>**Normal exposure time:** 4 s<br>**Off time:** 1 s<br>**Bottom exposure time:** 30 s<br>**Bottom layers:** 2 + 1 transition<br>**Z-axis lift height:** 6 mm<br>**Z-axis lift speed**: 50 mm/min<br>**Z-axis retract speed:** 150 mm/min	**Compensation type:** Local<br>**Compensation min.:** 100 µm<br>**Z level min.:** 200 µm<br>**Compensation max.:** 450 µm<br>**Z level max.:** 1900 µm	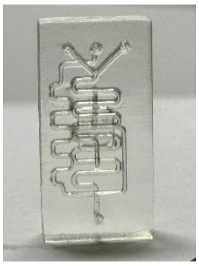<br>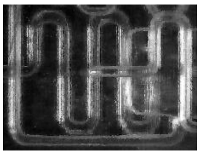
Mars 4	PB	**Layer thickness:** 0.05 mm<br>**Normal exposure time:** 2 s<br>**Off time:** 0.5 s<br>**Bottom exposure time:** 28 s<br>**Bottom layers:** 2 + 2 transitions<br>**Z-axis lift height:** 5 mm<br>**Z-axis lift speed**: 75 mm/min<br>**Z-axis retract speed:** 75 mm/min	**Compensation type:** Local<br>**Compensation min.:** 100 µm<br>**Z level min.:** 200 µm<br>**Compensation max.:** 400 µm<br>**Z level max.:** 1900 µm	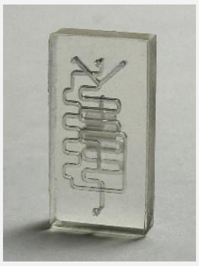<br>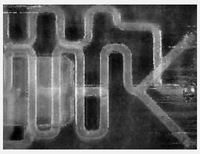

**Mars 4**	**BM**	**Layer thickness:** 0.05 mm<br>**Normal exposure time:** 3.5 s<br>**Off time:** 3 s<br>**Bottom exposure time:** 40 s<br>**Bottom layers:** 2 + 3 transitions<br>**Z-axis lift height:** 7 mm<br>**Z-axis lift speed:** 40 mm/min<br>**Z-axis retract speed:** 75 mm/min	**Compensation type:** Local<br>**Compensation min.:** 100 µm<br>**Z level min.:** 200 µm<br>**Compensation max.:** 450 µm<br>**Z level max.:** 1900 µm	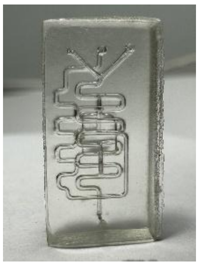 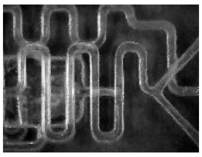
**Mars 4**	**BV**	**Layer thickness:** 0.05 mm<br>**Normal exposure time:** 1.8 s<br>**Off time:** 0.5 s<br>**Bottom exposure time:** 28 s<br>**Bottom layers:** 2<br>**Z-axis lift height:** 5 mm<br>**Z-axis lift speed**: 75 mm/min<br>**Z-axis retract speed:** 75 mm/min	**Compensation type:** Local<br>**Compensation min.:** 100 µm<br>**Z level min.:** 200 µm<br>**Compensation max.:** 400 µm<br>**Z level max.:** 2200 µm	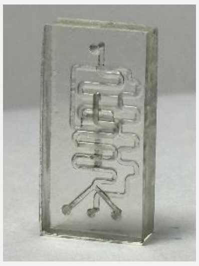 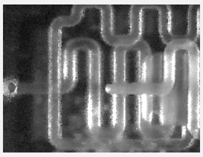

Table S2: Printer, slicing and DFM settings for each additional validation experiment.

1) Abbreviations stand for: D2 – Anycubic Photon D2, Mars 4 – Elegoo Mars 4.

2) Abbreviations stand for: BV – Miicraft BV-007A, PB – Anycubic Plant Based + Clear, BM – Liqcreate Bio-Med Clear.

Supplementary Note 3: Design and Printing Instructions

When designing microfluidic devices, it's important to position the feature layer(s) as close to the bottom (0 µm) as possible to minimize exposure to any remaining resins. To achieve this, it's best to print the side (top) with port openings facing downward towards the build plate, leaving the bottom layer to be printed last.

However, this orientation may lead to issues with ports being sealed if the base layers are exposed for a longer duration than subsequent layers. To mitigate this potential problem, Flui3d's ports feature incorporates a chamfer, which can be customized in size through the Property Inspector. Adjusting the radius and depth of the chamfer ensures that the ports remain open and unsealed, addressing any concerns about sealing during the printing process.

Supplementary Note 4: DFM Compensation Settings

Three-Layer Three-Way Mixer:

Compensation type: Local
Compensation min.: 100 µm
Z level min.: 200 µm
Compensation max.: 400 µm
Z level max.: 1900 µm

Mini. Protein Immunoassay:

Compensation type: Local
Compensation min.: 0 µm
Z level min.: 0 µm
Compensation max.: 400 µm
Z level max.: 1000 µm

Mini. Genotoxic Evaluation:

Compensation type: Local
Compensation min.: 0 µm
Z level min.: 0 µm
Compensation max.: 400 µm
Z level max.: 1100 µm

Resistive Microfluidic Network:

Compensation type: Local
Compensation min.: 100 µm
Z level min.: 200 µm
Compensation max.: 400 µm
Z level max.: 1400 µm

Supplementary Note 5: Detailed Complexities

	SW	AC/IV	S3	FLUI3D
**PROTEIN IMMUNOASSAYS**				
STAGE 1				
**BASE**	19	13	16	17
STAGE 2 AND 3				
**2 PLANES**	18	16	14	0
20 channel segments (incl. 1 serpentine channel)	160	100	160	27
9 chambers	72	45	72	34
18 ports	108	72	90	46
2 vias	12	8	10	5
68 rounded corners	84	80	92	0
151 extrusion areas	172	163	172	0
STAGE 4				
**25 EXCLUSION BODIES**	0	0	32	0
**TOTAL COMPLEXITY:**	**645**	**497**	**658**	**129**
**RESISTIVE MICROFLUIDIC NETWORKS**				
STAGE 1				
**BASE**	19	13	16	17
STAGE 2 AND 3				
**2 PLANES**	18	16	14	0
45 channel segments (incl. 6 serpentine channels)	360	225	360	71
2 ports	12	8	10	3
10 vias	60	40	50	12
57 extrusion areas	78	69	78	0
STAGE 4				
**16 EXCLUSION BODIES**	0	0	23	0
**TOTAL COMPLEXITY:**	**547**	**371**	**551**	**103**
**GENOTOXIC EVALUATION**				
STAGE 1				
**BASE**	19	13	16	17
STAGE 2 AND 3				
**2 PLANES**	18	16	14	0
15 channel segments	120	75	120	29
3 chambers	24	15	24	29
18 ports	108	72	90	32
3 vias	18	12	15	6
12 rounded corners	16	15	18	0
39 extrusion areas	60	51	60	0
STAGE 4				
**31 EXCLUSION BODIES**	0	0	38	0
**TOTAL COMPLEXITY:**	**383**	**269**	**395**	**113**

SARS-COV-2 ANTIBODY DETECTION				
		STAGE 1		
**BASE**	19	13	16	17
		STAGE 2 AND 3		
**1 PLANE**	9	8	7	0
197 channel segments (incl. 12 serpentine channels)	1576	985	1576	326
17 chambers	136	85	136	76
8 ports	64	40	64	36
13 transitions (wide)	260	208	195	85
29 transitions (height)	841	696	638	94
80 rounded corners	84	83	86	21
264 extrusion areas	523	412	523	0
		STAGE 4		
**106 EXCLUSION BODIES**	0	0	113	0
**TOTAL COMPLEXITY:**	**3512**	**2530**	**3354**	**655**
**3D DROPLET GENERATOR**				
		STAGE 1		
**BASE**	28	13	16	17
		STAGE 2 AND 3		
**2 PLANES**	18	16	14	0
4 channel segments	32	20	32	14
3 ports	18	12	15	6
1 via	6	4	5	3
8 extrusion areas	29	20	15	0
		STAGE 4		
**8 EXCLUSION BODIES**	0	0	15	0
**TOTAL COMPLEXITY:**	**131**	**85**	**112**	**40**
**ACTIVE FLOW CONTROL**				
		STAGE 1		
**BASE**	19	13	16	17
		STAGE 2 AND 3		
**1 PLANE**	9	8	7	0
4 channel segments	32	20	32	9
2 chambers	16	10	16	10
5 ports	30	20	25	6
10 rounded corners 3	22	19	28	5
14 extrusion areas	28	22	21	0
		STAGE 4		
**6 EXCLUSION BODIES**	0	0	13	0
**TOTAL COMPLEXITY:**	**156**	**112**	**158**	**47**
**PLANAR DROPLET GENERATOR**				
		STAGE 1		
**BASE**	19	13	16	17

STAGE 2 AND 3				
**1 PLANE**	9	8	7	0
62 channel segments (incl. 3 serpentine channels)	496	310	496	36
**3 PORTS**	18	12	15	4
96 rounded corners	104	102	102	0
66 extrusion areas	80	74	73	0
STAGE 4				
**4 EXCLUSION BODIES**	0	0	11	0
**TOTAL COMPLEXITY:**	**726**	**519**	**720**	**57**

Table S1: Detailed Complexities. Abbreviations stand for: SW – SolidWorks, AC - AutoCAD, IV - Autodesk Inventor, S3 -Shapr3D

Supplementary Note 6: Instructions for Utilizing the Flui3d Reference Design Model to Determine Optimal Compensation Settings

Procedure:

1. Download the Flui3d reference design model file and open it using the Flui3d software.
2. Familiarize yourself with the reference design model, which consists of nine feature parts, each having a different height ranging from 200 to 1000 microns. Note that all channels in the model have a width of 400 microns.
3. Modify the reference design model according to the smallest channel width present in your actual design, ensuring compatibility with your specific requirements.
4. Adjust the height of the single layer in the reference design model by setting it to position Z, where Z equals 4000 minus the position of the highest layer in your actual design. For example, if your design has four layers and the highest layer is at 1800 microns, set the layer height of the reference design model to 2200 microns (4000-1800).
5. Prepare the reference design model for printing by placing the ports facing down, ensuring they are printed last.
6. Proceed with printing the reference design model.
7. After printing, thoroughly wash and clean the printed model.
8. Inspect the printed model and identify the part with the next closest channel height that can be cleaned and is not fully cured, considering the smallest height of channels and components in your actual design.
9. Calculate the compensation value by subtracting the channel and component height of your actual design from the height of the channel of the identified part. This value will serve as the optimal "max" compensation setting (C_{max} at Z_{max}, where Z_{max} is the position of the highest layer in your actual design) for your printer setup.
10. For the "min" compensation setting (C_{min} at Z_{min}), it is recommended to use 0 for both values. However, you are free to fine-tune this value to add additional compensation as needed.

a. b.

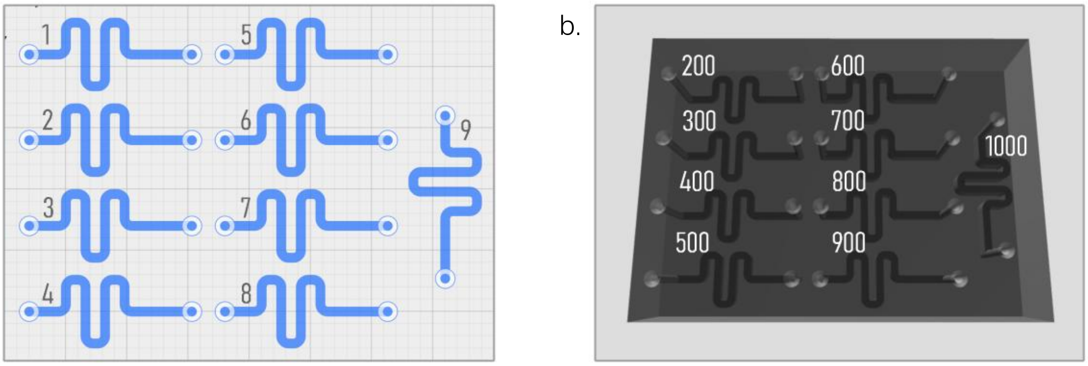

Figure S1: The reference design model with nine feature parts, each having a different height ranging from 200 to 1000 microns. (a) Nine feature parts and (b) their corresponding height.

Supplementary Methods: Flui3d's Software Design and Architecture

Overview

Unlike traditional EDA software, Flui3d is an open-source web-based application that offers a WYSIWYG (What You See Is What You Get) design interface. Users can effortlessly access the web-based design platform without the need to install any software. Flui3d employs a monolithic architecture comprising two key components: the frontend and backend. The frontend handles all design aspects, while the backend generates STL files. The following diagram illustrates the workflow.

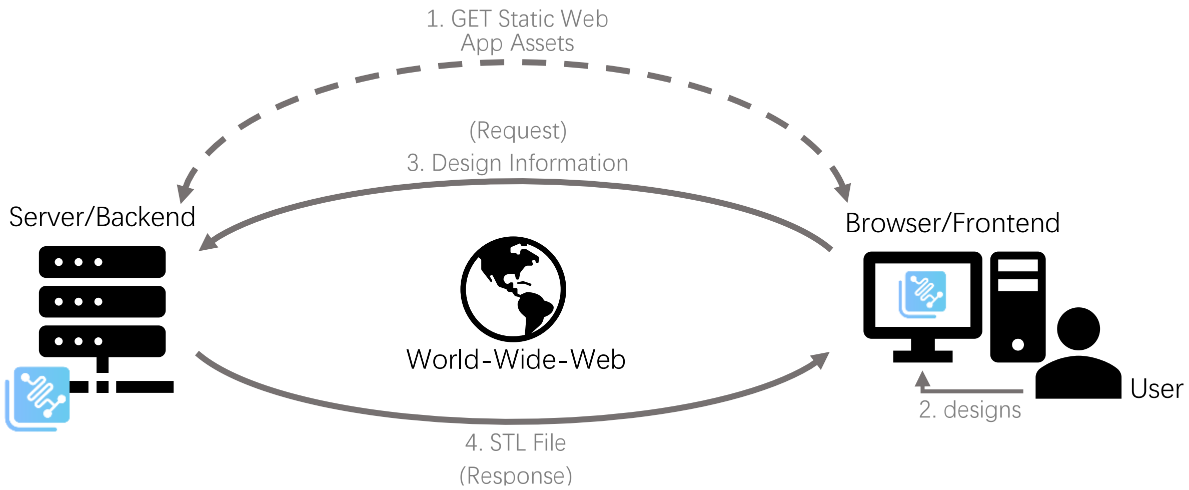

Figure S2: Workflow of Flui3d.

Frontend

The frontend operates as a fully static environment, executing all applications and business processes locally on the client/browser side. It is developed in TypeScript and leverages Vue 3 as its primary framework. The UI design is predominantly crafted using HTML and CSS, with Bootstrap 5.2 providing additional support.

Every UI element, as well as components from our Standard Parameterized Component Library, is defined as Vue components. The UI logic is coded in TypeScript. All graphical representations, including shapes and microfluidic components on the screen, are defined in Scalable Vector Graphics (SVG).

The frontend communicates with the backend in an asynchronous manner, transmitting the design information created by the user. The design information encompasses geometric details such as shape, dimensions, and location. Subsequently, the frontend receives the STL data generated by the backend and presents it to the user.

After building the code, a purely static HTML with resource hints injection is generated with all other assets including libraries. These files can be hosted on any web server and serve as the frontend. The diagram below represents the overall structure.

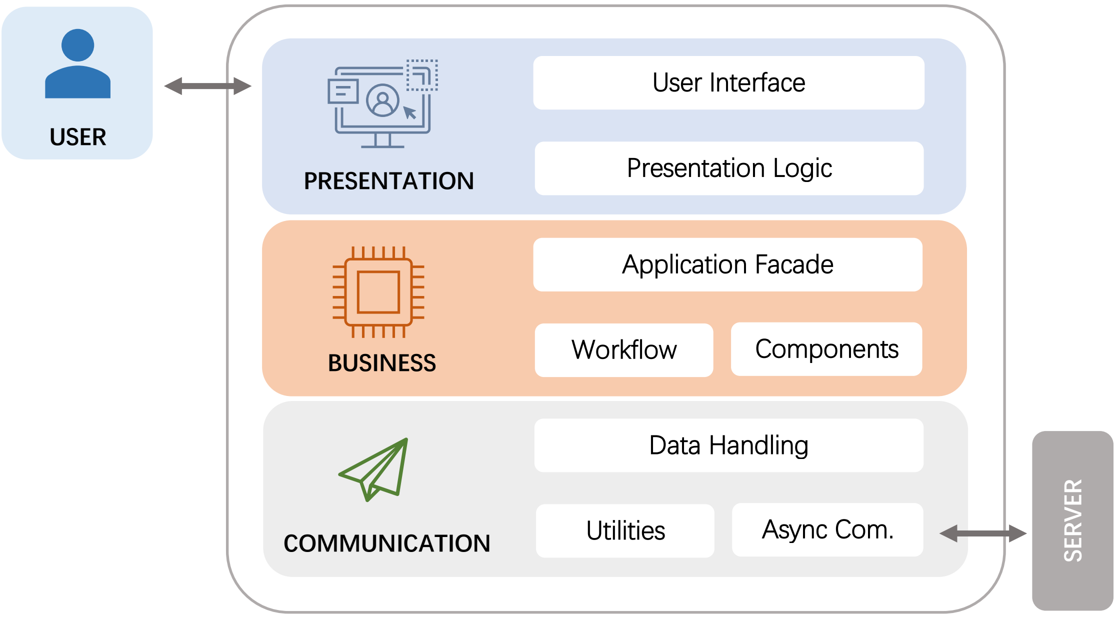

Figure S3: Overall structure of Flui3d's frontend.

Backend

The backend is exclusively implemented in Java and is composed of two crucial components: the servlet and the business logic. The servlet manages incoming requests and passes relevant information to the business logic. Within the business logic, a parser interprets the incoming data and invokes corresponding methods to construct the requested shapes as facets. Subsequently, the facets are transformed into STL data content and sent back to the servlet, which, in turn, responds to the originating source.

We have abstracted various shapes integral to microfluidic design into the following primitive types and classes: cuboid, cylinder, chamfer, torus, square ring, and polygon prism. Microfluidic components, such as channels or chambers, are created by combining these primitive types. The generation process follows the application of the factory design pattern.

The backend can be hosted using Tomcat. The following diagram illustrates the overall structure.

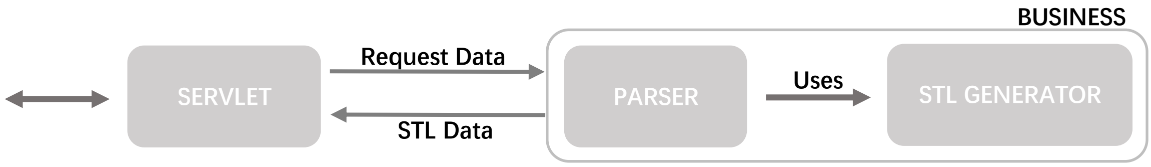

Figure S4: Overall structure of Flui3d's backend.
