# Supplementary material for: Open-source interactive design platform for 3D-printed microfluidic devices
Source: Commun Eng. 2024 May 18;3:71. doi: 10.1038/s44172-024-00217-0 (PMC11102439; doi:10.1038/s44172-024-00217-0)
Supplement: Supplementary file 3 — Description of Additional Supplementary Files [file 44172_2024_217_MOESM3_ESM.pdf]

# Description of Additional Supplementary Files

**File name:** Supplementary Data 1

**Description:** 1. 3D_Droplet.json Flui3d design file for the 3D droplet generator.

2. Array Geno.json Flui3d design file for the genotoxic evaluation.

3. Bridge_Example.json Flui3d design file for the bridge example.

4. Covid_Antibody.json Flui3d design file for the SARS-CoV2 antibody detection.

5. Droplet.json Flui3d design file for the planar droplet generator.

6. Geno_Miniaturized.json Flui3d design file for the genotoxic evaluation (miniaturized).

7. Pamicon.json Flui3d design file for the active flow control.

8. Protein_Miniaturized.json Flui3d design file for the protein immunoassays (miniaturized).

9. Protein.json Flui3d design file for the protein immunoassays.

10. Ref_Desgin_Model.json Flui3d design file of the reference design model.

11. RMN.json Flui3d design file for the resistive microfluidic networks.

12. Three_way_mixer.json Flui3d design file of the threelayer three-way mixer.
